# The Relationship Between Esophageal Motility Disorders and Varicella Zoster Virus: A Study Using Salivary DNA

**DOI:** 10.1007/s10620-025-09309-z

**Published:** 2025-08-11

**Authors:** Satsuki Takahashi, Tomoaki Matsumura, Michiko Sonoda, Tatsuya Kaneko, Ryosuke Horio, Chihiro Goto, Akane Kurosugi, Tsubasa Ishikawa, Yuki Ohta, Takashi Taida, Masato Nakamura, Kenichiro Okimoto, Jun Kato

**Affiliations:** Department of Gastroenterology, Graduate School of Medicine, Chiba University, Inohana 1-8-1, Chiba City, 260-8670 Japan

**Keywords:** Esophageal motility disorders, Nested PCR, Salivary DNA, Varicella zoster virus

## Abstract

**Background:**

The causes of most esophageal motility disorders (EMDs) remain unknown. Recent reports have suggested a potential association between the EMD achalasia and varicella zoster virus (VZV) reactivation. This study aimed to investigate the relationship between various EMDs and VZV infection.

**Methods:**

This was cross-sectional study. The participants in this study underwent high-resolution manometry (HRM) at Chiba University Hospital between 2022 and 2024. Saliva and blood samples were collected from all patients. The presence of VZV DNA in the saliva was determined using nested polymerase chain reaction (PCR) and electrophoresis. VZV-immunoglobulin M and immunoglobulin G antibody titers were measured in the blood samples. Based on the HRM results, the patients were divided into an EMD group and a normal group. The results were compared between these groups.

**Results:**

A total of 72 patients were included. Of these, 32 (44.4%) were in the normal group and 40 (55.6%) were in the EMD group, including 22 with esophagogastric junction (EGJ) outflow disorders (achalasia and EGJ outflow obstruction) and 18 with peristalsis disorders (absent contractility, distal esophageal spasm, hypercontractile esophagus, and ineffective esophageal motility). One patient with absent contractility had a concurrent herpes zoster infection, and VZV DNA was detected in this patient’s saliva. However, all other patients tested negative for VZV DNA. No significant difference in VZV antibody titers was observed between the two groups.

**Conclusions:**

VZV DNA in the saliva and VZV antibody titers do not appear to be associated with EMDs.

**Supplementary Information:**

The online version contains supplementary material available at 10.1007/s10620-025-09309-z.

## Introduction

Esophageal motility disorders (EMDs) encompass a heterogeneous group of conditions characterized by abnormalities in esophageal peristalsis and lower esophageal sphincter (LES) function. Symptoms may include dysphagia, acid reflux, and chest pain. These disorders frequently coexist with gastroesophageal reflux disease (GERD) and, in some cases, GERD may both result from and contribute to an EMD [1]. Secondary EMDs can arise from malignancies, surgical procedures, scleroderma, and myotonic dystrophy [2–4]. In contrast, primary EMDs such as idiopathic achalasia and spastic esophageal disorders have unclear etiologies. Achalasia is characterized by the progressive loss of esophageal peristalsis and failure of LES relaxation. This is linked to the loss of ganglion cells within Auerbach’s plexus, along with the depletion of key neurotransmitters, including vasoactive intestinal peptides and nitric oxide [5, 6]. Recent studies have implicated the varicella zoster virus (VZV) in the pathogenesis of achalasia. Naik et al. detected VZV deoxyribonucleic acid (DNA) and transcripts encoding late viral gene products in the esophageal tissue of patients with achalasia, suggesting that viral reactivation in esophageal neurons may contribute to dysmotility [7].

VZV is a neurotropic DNA virus of the herpes family. Following primary infection, it establishes lifelong latency in the sensory ganglia [8]. Reactivation of VZV in immunocompromised individuals leads to herpes zoster. Salivary detection of VZV DNA is recognized as a potential diagnostic marker for herpes zoster and enteric zoster [9, 10]. However, the relationship between EMDs and VZV remains unclear, and, to date, no studies have assessed the prevalence of salivary VZV DNA in EMD patients. This study aimed to investigate the presence of salivary VZV DNA in patients with EMD and to evaluate its potential association with EMD pathogenesis.

## Methods

### Study Design and Participants

This cross-sectional study was conducted at Chiba University Hospital. Patients who underwent high-resolution manometry (HRM) to test for esophageal abnormalities between May 2022 and July 2024 were recruited. Patients who did not consent to the study and those under 20 years of age were excluded. All participants provided saliva and blood samples and completed a questionnaire about their history of varicella, herpes zoster, and VZV vaccination. Patients with active herpes zoster were excluded from the main analysis.

Based on the HRM results, patients were categorized into a normal group and an EMD group. The EMD group was further stratified into those with esophagogastric junction (EGJ) outflow disorders (achalasia and EGJ outflow obstruction [EGJOO]) and those with peristalsis disorders (absent contractility, distal esophageal spasm, hypercontractile esophagus, and ineffective esophageal motility). In patients with EGJ outflow disorders, symptom onset was classified as either acute (developing abruptly within two months) or insidious [11].

This study was approved by the Medical Ethics Board of Chiba University Graduate School of Medicine (protocol no. M10194) and was conducted in accordance with the 1964 Declaration of Helsinki and its later revisions. Written informed consent to study participation was obtained from each patient.

### High-Resolution Manometry

HRM was conducted using BioView® v. 5.7.1.0 (formerly by Sandhill Scientific Inc., now by Diversatek Inc., Milwaukee, WI, USA) and Eight Star® v. 8.1-22.5 (Star Medical Inc., Tokyo, Japan) software. The manometry catheter was also manufactured by Diversatek Inc. All patients underwent overnight fasting prior to the procedure. During the HRM, each patient swallowed 10 × 5 ml of water in succession while in the supine position. This was followed by a multiple rapid swallow sequence of five consecutive swallows. They then swallowed an additional 10 × 5 ml of water in the upright position. EMDs were diagnosed based on the Chicago Classification system, v. 4.0 [12]. However, in cases where HRM was performed as a post-treatment evaluation of previously diagnosed EMDs and the results demonstrated a change between the pre-operative and post-operative state, we used the results of their initial pre-operative HRM in our analysis.

### Sample Collection

Saliva samples were obtained immediately before the HRM procedure, with at least 2 ml collected from each participant after a minimum fasting period of 30 min using the passive drool method into polypropylene tubes. For patients who had been previously diagnosed with achalasia based on prior HRM testing, saliva samples could not be collected immediately before the procedure and were instead obtained during the follow-up period. The samples were subsequently stored at − 30 °C until needed. Prior to DNA extraction, the samples were centrifuged at 2,000 × g for 5 min. The DNA was then extracted using the QIA-Amp® DNA Mini Kit (Qiagen, Inc., Chatsworth, CA, USA) in accordance with the manufacturer’s protocol. Nested polymerase chain reactions (PCRs) targeting open reading frame (ORF) 38 were conducted using TaKaRa Ex Premier™ DNA Polymerase (Takara Bio Inc., Shiga, Japan) per the manufacturer’s instructions. The DNA extracted from the saliva was initially amplified with external primers (30 cycles, denaturation at 98 °C for 10 s, annealing at 55 °C for 15 s, and extension at 68 °C for 19 s). Subsequently, 1.0 µl of the resulting amplicons were subjected to a second round of amplification with internal primers (30 cycles, denaturation at 98 °C for 10 s, annealing at 55 °C for 15 s, and extension at 68 °C for 15 s). The external primers used were TTC AGC CAA CGT GCC AAT AAA (forward) and GAC GCG CTT AAC GGA AGT AAC (reverse). The internal primers used were CTT GAT CCG TGT CAT CAT CAC (forward) and CTG ATG TGG TTA CGG AAG ACG (reverse). These primers were designed based on sequences published by the National Institute of Infectious Diseases [13]. The PCR products were electrophoresed on 1.5% agarose gel. The detection sensitivity of this assay ranged from 5 to 10 copies/μl. Additionally, serum samples were collected from blood specimens to measure VZV-immunoglobulin M (IgM) and VZV-immunoglobulin G (IgG) antibody titers using an enzyme immunoassay.

### Statistical Analysis

Baseline data were expressed as the median (interquartile range) or number (percentage). Continuous variables were analyzed using the Mann–Whitney U test. *P*-values < 0.05 were considered statistically significant. All statistical analyses were conducted using the SPSS for Windows software, v. 29 (IBM Corp., Armonk, NY, USA).

## Results

### Patient Characteristics and VZV DNA Detection

**Fig. 1:**
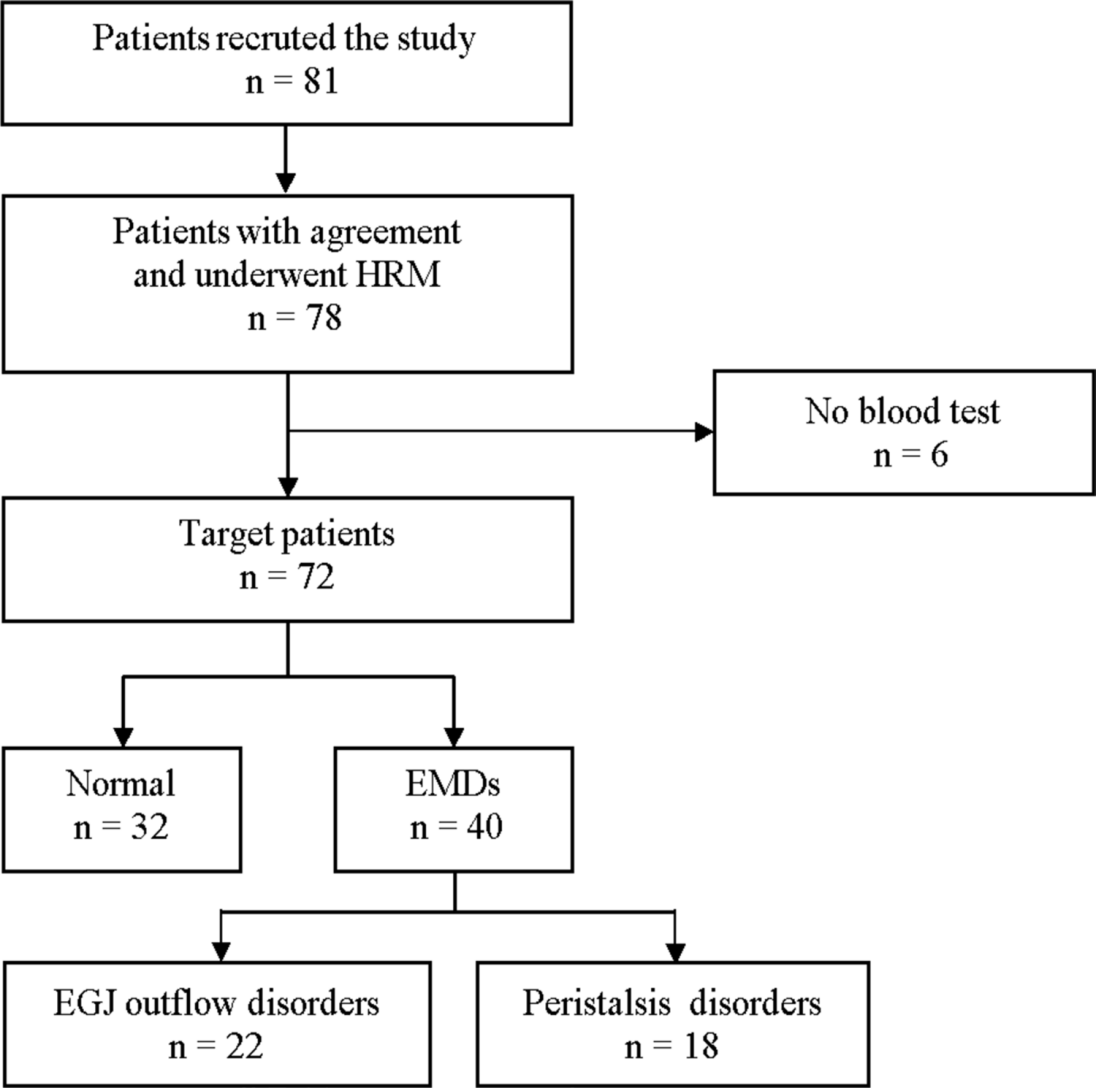
Flowchart of patient enrolment.

Of the 78 patients who underwent HRM, six were excluded due to missing blood test data (Fig. 1). A total of 72 patients were included in our analysis, of whom, 40 (55.6%) were male. The median age was 59 years (Table 1). The primary indications were as follows: dysphagia or suspected achalasia in 21 patients (29.2%), non-cardiac chest pain in 15 (20.8%), GERD symptoms in 14 (19.4%), laryngopharyngeal reflux symptoms in 9 (12.5%), follow-up after peroral endoscopic myotomy (POEM) or pneumatic dilation in 4 (5.6%), and other indications in 9 (12.5%). The normal group comprised 32 (44.4%) of the sample. The remaining 40 (55.6%) were in the EMD group. The EMD group included 22 patients with EGJ outflow disorders and 18 with peristalsis disorders. Four patients with previously diagnosed achalasia had already undergone POEM or pneumatic dilation, and three of them were classified as having absent contractility in the current HRM. These patients were categorized as achalasia according this study protocol. The median interval between the HRM procedure and saliva collection was 0 days (interquartile range: 0–0 days). One patient was 5 days post-herpes zoster onset. The median salivary DNA concentration was 11.1 ng/μl. All salivary VZV DNA PCR results were negative except for the herpes zoster patient. All patients tested positive for VZV-IgG (≥ 2.0), suggesting prior VZV exposure or vaccination.

**Table 1:** Patient characteristics and results from their varicella zoster virus DNA and varicella zoster virus antibody tests

All patients	*N* = 72
Male sex, (%)	40 (55.6)
Age, years, median (IQR)	59 (47–74)
HRM result, (%)	
Normal	32 (44.4)
Disorders of EGJ outflow	22 (30.6)
Achalasia	19 (26.4)
EGJOO	3 (4.2)
Disorders of peristalsis	18 (25)
Absent contractility	4 (5.6)
Distal esophageal spasm	2 (2.8)
Hypercontractile esophagus	2 (2.8)
Ineffective esophageal motility	10 (13.9)
Infection history	
Varicella (+ / − /unknown)	26/21/25
Herpes zoster (+ / − /unknown)	20/43/9
VZA vaccination history (+ / − /unknown)	9/46/17
DNA concentration (ng/μL), median (IQR)	11.1 (6.2–24)
Positive VZV DNA, (%)	1 (1.4)
VZV-IgM titers, median (IQR)	0.26 (0.23–0.30)
VZV-IgG titers, median (IQR)	14 (8.3–22)

*DNA* deoxyribonucleic acid, *EGJ* esophagogastric junction, *EGJOO* esophagogastric junction outflow obstruction, *HRM* high-resolution manometry, *IgG* immunoglobulin G, *IgM* immunoglobulin M, *IQR* interquartile range, *VZV* varicella zoster virus

### Results of the Herpes Zoster Patient

The patient with herpes zoster was an 84-year-old woman with absent contractility (Table 2). She tested positive for salivary VZV DNA. Her electrophoresis results confirmed the presence of VZV DNA (Suppl. Figure 1). The patient was positive for VZV-IgM and IgG antibodies. The titers for these were 6.8 and > 128, respectively.

**Table 2:** Details of the patient with active herpes zoster

Sex	Female
Age	84
HRM result	Absent contractility
Infection history	
Varicella	Unknown
Herpes zoster	Positive
VZV vaccination history	Negative
DNA concentration (ng/μL)	9.2
VZV-IgM titers	6.8
VZV-IgG titers	> 128

*DNA* deoxyribonucleic acid, *HRM* high-resolution manometry, *IgG* immunoglobulin G, *IgM* immunoglobulin M, *VZV* varicella zoster virus

### Comparison of Antibody Titers

Because herpes zoster patients have been reported to have VZV DNA in their saliva, we compared the saliva test results of the two groups, excluding the herpes zoster patient. We found no significant differences in VZV-IgM or IgG antibody titers between the EMD and normal groups (Table 3). Similarly, there were no significant differences between EGJ outflow disorders and peristalsis disorders, achalasia and non-achalasia, or acute onset and insidious onset (acute onset, 2; insidious onset, 17; unknown, 3) (Tables 3, 4, 5, Suppl. Table 1).

**Table 3:** Varicella zoster virus antibody titers in patients with esophageal motility disorders and normal controls

	EMDs	Normal	*P*-value
	*N* = 39	*N* = 32	
VZV-IgM titers, median (IQR)	0.26 (0.22–0.30)	0.26 (0.22–0.30)	0.835
VZV-IgG titers, median (IQR)	13.2 (8.0–22.4)	13.65 (8.2–21.23)	0.885

*EMD* esophageal motility disorder, *IgG* immunoglobulin G, *IgM* immunoglobulin M, *IQR* interquartile range, *VZV* varicella zoster virus

**Table 4:** Varicella zoster virus antibody titers between esophagogastric junction outflow disorders and peristalsis disorders

	EGJ outflow disorders	Peristalsis disorders	*P*-value
	*N* = 22	*N* = 17	
VZV-IgM titers, median (IQR)	0.25 (0.22–0.30)	0.26 (0.22–0.30)	0.989
VZV-IgG titers, median (IQR)	13.6 (9.2–22.6)	13.1 (7.2–22.5)	0.769

*EGJ* esophagogastric junction, *IgG* immunoglobulin G, *IgM* immunoglobulin M, *IQR* interquartile range, *VZV* varicella zoster virus

**Table 5:** Varicella zoster virus antibody titers between achalasia and others

	Achalasia	Others	*P*-value
	*N* = 19	*N* = 52	
VZV-IgM titers, median (IQR)	0.25 (0.22–0.29)	0.26 (0.22–0.30)	0.644
VZV-IgG titers, median (IQR)	13.6 (8.6–22.4)	13.4 (8.0–21.7)	0.928

*IgG* immunoglobulin G, *IgM* immunoglobulin M, *IQR* interquartile range, *VZV* varicella zoster virus

## Discussion

This study investigated the potential association between EMDs and VZV using salivary VZV DNA detection and serological antibody analysis. Our findings revealed no significant differences in either parameter between patients with EMDs and those with normal esophageal motility. Thus, we concluded that any relationship between EMDs and VZV was weak, negligible, or absent. However, given the complexity of EMD pathogenesis and previous reports indicating potential viral involvement, further investigation is warranted.

Esophageal achalasia is a primary motility disorder characterized by the degeneration of enteric neurons in the esophageal body and LES, the etiology of which is unclear. Previous studies have examined the possible role of viral infections, including VZV, in the pathogenesis of achalasia. Birgisson et al. employed PCR to detect viral DNA sequences in esophageal myotomy specimens from patients with achalasia, but found no viral DNA, including VZV, in either their experimental or control patients [14]. Similarly, Moradi et al. utilized PCR and reverse transcription PCR to amplify the DNA sequences of various viruses, including VZV, in esophageal biopsy tissue and blood specimens of patients with achalasia and controls. Again, both groups tested negative for VZV DNA [15]. These findings are consistent with our results.

Several studies have suggested a potential link between VZV and achalasia. In 1993, Robertson et al. reported a higher prevalence of VZV DNA in the esophageal myenteric plexus of patients with achalasia compared to controls, along with significantly elevated serum VZV antibody titers [16]. More recently, Naik et al. identified VZV DNA, transcripts, and proteins in esophageal muscularis specimens from achalasia patients and detected salivary VZV DNA in 12 of 15 achalasia patients [7]. Gaber et al. conducted a large case–control study using medical insurance claims data and found a significant association between VZV and achalasia [17]. However, salivary VZV DNA has been reported in individuals experiencing physiological stress, such as astronauts during or after space flight and medical residents on night calls [18, 19]. Maria et al. detected salivary VZV DNA in individuals up to 144 months post-herpes zoster infection, as well as some with no history of herpes zoster [20]. This suggests that salivary VZV DNA detection can be influenced by various physiological and environmental conditions. This is a considerable obstacle to attempts to evaluate the association between EMDs and viral infections. Future studies could provide further insights into the potential role of VZV in EMDs by approaching the musculature and nerves of the esophagus.

Aside from VZV, other viral pathogens, including herpes simplex virus 1 and SARS-CoV-2, have been implicated in esophageal neurodegeneration and achalasia pathogenesis [21]. Furuzawa-Carballeda et al. identified SARS-CoV-2 and its receptors in the LES muscle tissue of patients with type II achalasia following COVID-19 infection, but not in controls type II achalasia patients or transplant donors [22]. This suggests that viral infections may affect the integrity of the mesenteric plexus. However, achalasia is increasingly recognized as a multifactorial disorder, likely resulting from a complex interplay of genetic susceptibility, environmental triggers, allergy-mediated processes, and autoimmune-mediated neuronal degeneration [23, 24].

To the best of our knowledge, this is the first study to investigate salivary VZV DNA not only in achalasia but also in other EMD subtypes. EGJOO is regarded as a precursor or variant of achalasia [25], while absent contractility can be difficult to distinguish from type I achalasia [4]. However, our findings suggest that VZV is not strongly implicated in the pathogenesis of these disorders, further challenging the hypothesis that EMDs have direct viral etiologies.

Nevertheless, our study had some limitations. First, the sample size was relatively small. A larger cohort would provide more robust statistical results. Second, we utilized the primers suggested by the National Institute of Infectious Diseases for detecting Japanese wild-type VZV strains. However, prior studies investigating salivary VZV DNA have used PCR to target different ORFs [10, 26]. Although we validated our primer selection, alternative PCR conditions may yield additional results. Third, for patients in the normal group, endoscopic findings and multichannel intraluminal impedance-pH test results were not considered. Their HRM results were normal, but the final diagnosis may be some other disease. Finally, in patients with non-achalasia EMDs, associated symptoms and additional supportive testing (such as timed barium esophagram or EndoFLIP) were not considered in the analysis. Therefore, the clinical significance of non-achalasia EMDs remains unclear.

In conclusion, this study found no significant differences in the rates of salivary VZV DNA or VZV antibody titers between EMD patients and controls. Given the complex, multifactorial nature of EMDs, further research is warranted to explore alternative mechanisms that may underlie this group of disorders.

## Supplementary Information

Below is the link to the electronic supplementary material.

Supplementary file1 (DOCX 200 KB)

## Data Availability

The data that support the findings of this study are available from the corresponding author, TM (ORCID, 0000-0001-5314-9325), upon reasonable request.

## Abbreviations

DNA: Deoxyribonucleic acid
EGJ: Esophagogastric junction
EGJOO: Esophagogastric junction outflow obstruction
EMD: Esophageal motility disorders
GERD: Gastro-esophageal reflux disease
HRM: High-resolution manometry
LES: Lower esophageal sphincter
ORF: Open reading frame
PCR: Polymerase chain reaction
VZV: Varicella zoster virus
POEM: Peroral endoscopic myotomy
