# Supplementary material for: The Relationship Between Esophageal Motility Disorders and Varicella Zoster Virus: A Study Using Salivary DNA
Source: Dig Dis Sci. 2025 Aug 11;71(1):172–8. doi: 10.1007/s10620-025-09309-z (PMC12909326; doi:10.1007/s10620-025-09309-z)
Supplement: Supplementary file 1 — Supplementary file1 (DOCX 200 KB) [file 10620_2025_9309_MOESM1_ESM.docx]

**Supplementary materials**

**Suppl. Table 1. Varicella zoster virus antibody titers of patients with acute onset and insidious onset esophageal motility disorders**

|  | Acute onset | Insidious onset |  |
| --- | --- | --- | --- |
|  | N = 2 | N = 17 | *p*-value |
| VZV-IgM titers, median (IQR) | 0.27 (0.21–*) | 0.25 (0.24–0.30) | 0.947 |
| VZV-IgG titers, median (IQR) | 16.6 (10.8–*) | 13.5 (7.4–19.9) | 0.842 |

*The third quartile did not exist. IgG, immunoglobulin G; IgM, immunoglobulin M; IQR, interquartile range; VZV, varicella zoster virus

**Suppl. Fig. 1. Electrophoresis gel result from a patient with herpes zoster**


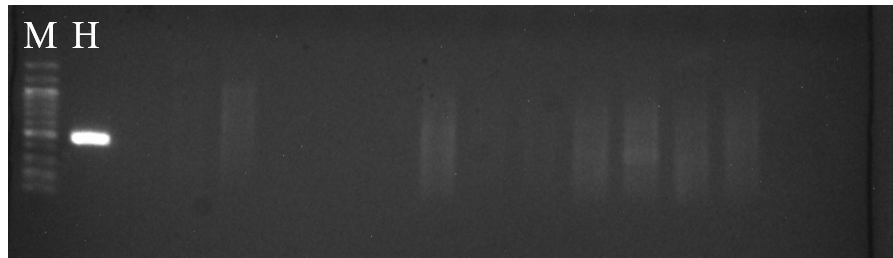


Representative gel after nested PCR for salivary VZV-DNA in 16 samples. (H) is a sample from a herpes zoster patient (498 mer).

DNA, deoxyribonucleic acid; H, herpes zoster sample; M, measurement; PCR, polymerase chain reaction; VZV, varicella zoster virus
